# Molecular Characterization of Striated Muscle-Specific Gab1 Isoform as a Critical Signal Transducer for Neuregulin-1/ErbB Signaling in Cardiomyocytes

**DOI:** 10.1371/journal.pone.0166710

**Published:** 2016-11-18

**Authors:** Taku Yasui, Takeshi Masaki, Yoh Arita, Tomohiko Ishibashi, Tadakatsu Inagaki, Makoto Okazawa, Toru Oka, Wataru Shioyama, Keiko Yamauchi-Takihara, Issei Komuro, Yasushi Sakata, Yoshikazu Nakaoka

**Affiliations:** 1 Department of Vascular Physiology, National Cerebral and Cardiovascular Center Research Institute, Suita, Osaka, Japan; 2 Department of Cardiovascular Medicine, Osaka University Graduate School of Medicine, Suita, Osaka, Japan; 3 Department of Cardiology, Osaka Medical Center for Cancer and Cardiovascular Diseases, Osaka, Japan; 4 Department of Cardiovascular Medicine, The University of Tokyo Graduate School of Medicine, Tokyo, Japan; 5 Precursory Research for Embryonic Science and Technology (PRESTO), Japan Science Technology Agency, Kawaguchi, Saitama, 332-0012, Japan; Cincinnati Children's Hospital Medical Center, UNITED STATES

## Abstract

Grb2-associated binder (Gab) docking proteins regulate signals downstream of a variety of growth factors and receptor tyrosine kinases. Neuregulin-1 (NRG-1), a member of epidermal growth factor family, plays a critical role for cardiomyocyte proliferation and prevention of heart failure via ErbB receptors. We previously reported that Gab1 and Gab2 in the myocardium are essential for maintenance of myocardial function in the postnatal heart via transmission of NRG-1/ErbB-signaling through analysis of Gab1/Gab2 cardiomyocyte-specific double knockout mice. In that study, we also found that there is an unknown high-molecular weight (high-MW) Gab1 isoform (120 kDa) expressed exclusively in the heart, in addition to the ubiquitously expressed low-MW (100 kDa) Gab1. However, the high-MW Gab1 has been molecularly ill-defined to date. Here, we identified the high-MW Gab1 as a striated muscle-specific isoform. The high-MW Gab1 has an extra exon encoding 27 amino acid residues between the already-known 3^rd^ and 4th exons of the ubiquitously expressed low-MW Gab1. Expression analysis by RT-PCR and immunostaining with the antibody specific for the high-MW Gab1 demonstrate that the high-MW Gab1 isoform is exclusively expressed in striated muscle including heart and skeletal muscle. The ratio of high-MW Gab1/ total Gab1 mRNAs increased along with heart development. The high-MW Gab1 isoform in heart underwent tyrosine-phosphorylation exclusively after intravenous administration of NRG-1, among several growth factors. Adenovirus-mediated overexpression of the high-MW Gab1 induces more sustained activation of AKT after stimulation with NRG-1 in cardiomyocytes compared with that of β-galactosidase. On the contrary, siRNA-mediated knockdown of the high-MW Gab1 significantly attenuated AKT activation after stimulation with NRG-1 in cardiomyocytes. Taken together, these findings suggest that the striated muscle-specific high-MW isoform of Gab1 has a crucial role for NRG-1/ErbB signaling in cardiomyocytes.

## Introduction

Neuregulin-1 (NRG-1), a member of the epidermal growth factor (EGF) family, serves as a paracrine factor that is shed from the endocardial and capillary endothelial cell in the heart, and exerts various effects via the erythroblastic leukemia viral oncogene homolog (ErbB) 2, 3, and 4 receptor tyrosine kinases (ErbB2, ErbB3, and ErbB4) [1–3]. Among these ErbB receptors, NRG-1 activates the ErbB4 homodimer or ErbB2/ErbB4 heterodimer expressed on cardiomyocytes, and plays critical roles in both heart development and cardiac homeostasis [1–5]. NRG-1-, ErbB2- and ErbB4-knockout (KO) mice display embryonic lethality and similar defects in ventricular trabeculation [6–8]. The importance of ErbB signaling in human adult heart was revealed by the unforeseen adverse effects of trastuzumab (Herceptin), a monoclonal antibody against ErbB2 widely used for the treatment of breast cancer. Trastuzumab induces heart failure when combined with anthracycline treatment [2,9,10]. Consistent with the clinical evidence, cardiomyocyte-specific ErbB2- and ErbB4-KO mice both exhibit dilated cardiomyopathy (DCM) phenotype in adulthood [11–13].

An EGF-domain fragment of recombinant human (rh) NRG-1 has been shown to have a significant effect on heart function and survival in a series of small and large animal models of systolic heart failure [14]. Furthermore, NRG-1 induces cardiomyocyte proliferation via phosphatidylinositol 3-kinase (PI3-kinase) pathway and contributes to repair after myocardial ischemic injury in rodents [15]. However, the molecular mechanism how PI3-kinase/AKT signaling axis is activated downstream of NRG-1/ErbB signaling has not been fully elucidated.

Grb2-associated binder (Gab) family docking proteins, consisting of Gab1, Gab2 and Gab3, are involved in amplification and integration of signal transduction induced by growth factors and cytokines [16–18]. Gab family proteins undergo tyrosine-phosphorylation upon stimulation and associate with Src holomology-2 (SH2) domain-containing proteins such as protein phosphatase SHP2, PI3-kinase regulatory subunit p85, phospholipase Cγ, Crk, and GC-GAP. Docking of Gab proteins to SHP2 and p85 is considered to be essential for activation of mitogen activated kinase extracellular signal-regulated kinase (ERK)1/2, and AKT, respectively [16,17].

Conventional Gab1 knockout (Gab1KO) mice display embryonic lethality with impaired development of heart, placenta, skin and skeletal muscle [19,20]. Because Gab1KO mice show embryonic lethality, several groups including ours created conditional knockout mice of Gab1 using the *Cre-loxP* system [21–30]. We and other group created cardiomyocyte-specific *Gab1*-knockout (Gab1CKO) mice, but these mice are viable and display no obvious cardiac phenotypes under normal condition [22,27]. On the other hand, cardiomyocyte-specific *Gab1/Gab2* double knockout (DKO) mice showed a high postnatal mortality rate with marked ventricular dilatation and reduced contractility due to the defects in NRG-1/ErbB signaling [22].

In the previous study, we found that a novel high-molecular weight (high-MW) Gab1 (120–130 kDa) was expressed exclusively in the heart in addition to the low-MW Gab1 (100 kDa), an already-known ubiquitously expressed isoform [22]. The high-MW Gab1 underwent tyrosine-phosphorylation exclusively upon stimulation with NRG-1 among several growth factors and cytokines, suggesting that the high-MW Gab1 isoform might have a role for NRG-1-dependent signaling in cardiomyocytes [22]. However, the molecular character of the high-MW Gab1 has been elusive to date. Here we identified a novel high-MW Gab1 which is exclusively expressed in striated muscle, and characterized it as a crucial signaling component for the activation of PI3-kinase/AKT signaling axis downstream of NRG-1/ErbB signaling in cardiomyocytes.

## Materials and Methods

### Animals

All experiments were carried out under the guidelines of the Osaka University Committee for Animal and Recombinant DNA Experiments and the local Animal Ethics Committee of the National Cerebral and Cardiovascular Center Research Institute (Osaka). All experiments were also approved by the Institutional Review Board of Osaka University and the National Cerebral and Cardiovascular Center Research Institute. Male C57BL/6J 8-week(wk)-old mice and timed-pregnant C57BL/6J mice, purchased from Japan SLC, Inc., were used in the experiments.

### Reagents

Anti-high-MW Gab1 antiserum was generated by immunizing rabbits with the peptide (CQGSSFVSEEGEEYLLLEDFESKTIPLQ) encoded by the human Gab1 3–4 extra exon. For immunization, rabbits were first injected with 200 μg of the antigen in complete Freund’s adjuvant and then boosted every 2 wk with 100 μg of the antigen in incomplete Freund’s adjuvant. Other antibodies were purchased as follows: anti-phospho-Gab1 (Tyr627) (1:1000, #3231), anti-Gab1 (1:1000, #3232), anti-phospho-AKT (Ser473) (1:1000, #9271), anti-AKT (1:1000, #9272), anti-phospho-p44/p42 (pERK1/2) (1:1000, #9101), anti-p44/p42 (ERK1/2) (1:1000, #9102), anti-pErbB4 (Tyr1284) (1:1000, #4757) antibodies for immunoblotting, and horseradish peroxidase-coupled horse anti-mouse (1:3000, #7076) and goat anti-rabbit IgG (1:3000, #7074) from Cell Signaling Technology; antibodies recognizing phospho-tyrosine (PY99) (1:1000, sc-7020), ErbB4 (1:400, sc-283), and SHP2 (1:400, sc-280) were from Santa Cruz Biotechnology Inc; anti-Gab1 (1:1000, 06–579) antibody for immunoblotting of immunoprecipitated proteins, and anti-p85 (1:1000, 06–195) antibody were from Millipore; antibodies against β-tubulin (mouse monoclonal) (1:3000, T5201), and anti-α-actinin (1:300, A7732) were from Sigma-Aldrich. The anti-Gab1 serum for immunoprecipitation was used as described previously [22,23,31–33]. Medium 199 and BSA (cell culture grade) were purchased from Invitrogen. Collagenase, percoll, and recombinant NRG-1β (NRG-1β EGF domain; sold as heregulin-β1) were purchased from Sigma-Aldrich. HB-EGF and EGF were from R&D Systems. LIF was from Millipore.

### Immunoprecipitation and western blot analysis of mouse tissues

About 80 mg tissues were excised from male C57BL/6J 8-wk-old mouse and snap-frozen in liquid nitrogen. Frozen heart, brain, spleen, lung, liver, and skeletal muscle were homogenized in 50 mmol/L Hepes, 100 mmol/L sodium fluoride, 2 mmol/L sodium orthovanadate, 4 mmol/L EDTA, 1% Tween-20, 0.1% SDS, and Complete protease inhibitor cocktail (Roche Applied Science) using Polytron homogenizer (PT 10-35GT; Kinematica) as described previously [22,34]. The lysates were precleared by centrifugation at 15,000 × g for 15 minutes (min). For immunoprecipitation, the cleared lysates were rotationally incubated with either 1 μL of anti-Gab1 or 2 μL of anti-high MW Gab1 serum and with 20 μL of protein A-sepharose (GE Healthcare) for 8 hours (hr) at 4°C. The antigen-antibody complexes were collected by centrifugation, washed three times with lysis buffer without protease inhibitor mixture, and boiled in standard electrophoresis sample buffer. All the immunoprecipitated proteins were then resolved by SDS-PAGE and subjected to immunoblotting using standard procedure. Blots were developed using ECL system (GE Healthcare).

### Sequencing of mouse Gab1 Extra Exon

Total RNA was isolated from adult mouse heart, lung, and brain using TRIzol (Invitrogen). cDNA was synthesized using SuperScript II Reverse Transcriptase (Invitrogen) and subjected to PCR (94°C for 2 min; 25 cycles through 94°C for 30 seconds (sec), 60°C for 30 sec, 72°C for 90 sec, and then extension at 72°C for 10 min) using the forward primer 5’-CCTGTGAAGCCGCTGACTGGCTC-3’ located in exon 3 and the reverse primer 5’-TTTCCGCAACTTGTTCAAATCCACGG-3’ located in exon 4. PCR products were electrophoresed in a 1.5% agarose gel. Double bands were detected only in the cDNA purified from heart, and the higher band was purified using QIAEX II Agarose Gel Extraction Kit (QIAGEN). The purified PCR products were cloned using Zero Blunt TOPO PCR Cloning Kit for Sequencing (Invitrogen) and transformed into JM109 competent cells. Plasmid DNA was extracted and sequenced. The sequence was analyzed using BLAST.

### Cloning of the full-length mouse Gab1 cDNA

Total RNA was isolated from adult mouse heart using TRIzol. cDNA was synthesized using SuperScript II Reverse Transcriptase (Invitrogen) and subjected to PCR (94°C for 30 sec; 35 cycles through 94°C for 15 sec, 72°C for 3 min, and then extension at 72°C for 2 min) using the forward primer 5’-GCGGATCCATGAGCGGCGGCGAAGTG-3’ and the reverse primer 5’-CGGCGGCCGCTGGCTCGAGGCTCCACTGAGA-3’. Purified and restriction-digested products were inserted into pcDNA3 at BamHI and NotI restriction sites. pcDNA3 constructs of mouse Gab1 were transformed into JM109 competent cells. Plasmid DNA was extracted and subjected to PCR (94°C for 3 min; 30 cycles through 94°C for 30 sec, 60°C for 30 sec, 72°C for 40 sec, and then extension at 72°C for 4 min) using the forward primer 5’-CAAAGCAAGAAGCCTGAACC-3’ located in exon 3 and the reverse primer 5’-CTCTCCCGAGACAGATGTCAG-3’ located in exon 4. The predicted amplified fragments of mouse high- and low-MW Gab1 were 280 and 200 base pairs, respectively. Plasmid DNA which was thought to contain mouse high- or low-MW Gab1 was sequenced.

### Transfection and western blot analysis

pcDNA3 constructs of mouse high- or low-MW Gab1 was transfected into HEK293 cells with Lipofectamine 2000 according to the manufacturer’s instructions (Invitrogen). After 4 hr, the cells were returned to growth medium and incubated for 48 hr. Cells were washed twice with ice-cold PBS and scraped off in lysis buffer containing 20 mmol/L Tris (pH 7.4), 150 mmol/L NaCl, 3 mmol/L EDTA, 1% Nonidet P-40, 2 mmol/L orthovanadate and Complete protease inhibitor cocktail as described previously [22,23,32]. Cell lysates were precleared by centrifugation at 15,000 × g for 15 min. The cleared lysates were subjected to immunoblot analysis following standard procedures as described above.

### Expression analysis using cDNA panel

Mouse MTC panel I (Clontech) was utilized for the analysis of high-MW Gab1 expression pattern. The MTC cDNA was subjected to PCR (94°C for 3 min; 32 cycles through 94°C for 30 sec, 60°C for 30 sec, 72°C for 1 min, and then extension at 72°C for 7 min) using the forward primer 5’-CCAAGGGTCATCTTTTGTTTC-3’ located in extra exon or 5’-CAAAGCAAGAAGCCTGAACC-3’ located in exon 3 and the reverse primer 5’-CTCTCCCGAGACAGATGTCAG-3’ located in exon 4. PCR products were electrophoresed in a 1.5% agarose gel.

### Real-time Reverse Transcription-PCR

Total RNA from embryonic hearts and postnatal hearts was extracted using TRIzol reagent (Invitrogen). qRT-PCR was carried out using the QuantiFast SYBRGreen RT-PCR kit (Qiagen) as described previously[23,34,35]. For each reaction, 80 ng of total RNA was transcribed for 10 min at 50°C, followed by a denaturing step at 95°C for 5 min and 40 cycles of 10 s at 95°C and 30 s at 60°C. Fluorescence data were collected and analyzed using LightCycler 480 Instrument (Roche). The following primers were used: *Gapdh*: 5’-TCTCCACACCTATGGTGCAA-3’, 5’-CAAGAAACAGGGGAGCTGAG-3’; *Gab1*: 5’-AAATCTGTCTGGCGAAGACC-3’, 5’-TCTCCTTTGGGTTTATTCATCG-3’; *high-MW Gab1*: 5’-TTTGAAAGCAAAGCGATTCC-3’, 5’-TGCAGTCTGTCTCAGAAAAGGT-3’.

### Immunohistochemical analysis of mouse tissues

For immunohistochemial analysis, anti-high-MW Gab1 antiserum was absorbed with mouse liver and kidney powder as described previously [36]. Mouse liver and kidney powder was prepared from cold acetone homogenate [36]. Mouse liver and kidney powder was washed in PBS and used to absorb antiserum diluted 1:4 in 0.2% BlockAce (DS Pharma Biomedical). The mixture was shaken overnight at 4°C. The absorbed antiserum was separated from acetone powder by centrifugation at 10,000 × g for 10 min. Embryos were embedded in OCT (Sakura) for frozen sections and cut by cryosectioning (10 μm) [35]. Sections were fixed in pre-cooled acetone for 10 min. Sections were incubated in blocking solution containing 1% bovine serum albumin in PBS and 0.1% Triton-X (PBST) for 1 h at room temperature and then incubated with primary antibodies in PBST containing 1% bovine serum albumin overnight at 4°C. The following primary antibodies were used for immunostaining: absorbed anti-high MW Gab1 (1:10 dilution); anti-α-Actinin (1:300 dilution). Sections were then washed in PBST and incubated with fluorescent-conjugated (1:200 dilution; Alexa Fluor 488 and 546; Invitrogen) secondary antibodies for 1 hr at room temperature. Images were acquired with a confocal microscope (LSM710; Zeiss).

### Biochemical analysis in vivo

Biochemical analysis of mice hearts after stimulation with various growth factors and cytokines was performed as described previously [22]. Briefly, mice anesthetized by isoflurane were injected i.v. via the inferior vena cava with following agonists: LIF (1×10^4^ U), NRG-1β (5 μg), HB-EGF (5 μg), and EGF (5 μg). The hearts were isolated at 5 min after injection and washed with ice-cold PBS. After removing both atria, the ventricles were snap-frozen in liquid nitrogen. Immunoprecipitation and western blot analysis were performed as described above. Band intensity was quantified by densitometry.

### Adenovirus vector construction

The cDNA sequence encoding mouse high- and low-MW Gab1 was subcloned into the shuttle plasmid pACCMVpLpA. Recombinant adenovirus was then obtained according to the homologous recombination system described previously [32,37]. The adenovirus vector expressing β-gal was used as a control.

### Neonatal rat cardiomyocyte culture, adenovirus-mediated overexpression and siRNA-mediated knockdown experiments

Primary cultures of neonatal rat cardiomyocytes (NRCMs) were prepared from ventricles of 1- to 2-day-old Wistar rats (Kiwa Jikken Dobutsu and Japan SLC, Inc.) on Percoll gradient as described previously [22]. Briefly, ventricles were isolated from neonatal rats and treated with trypsin and collagenase for 30 min at 37°C. Isolated cells were suspended in 58.5% Percoll in HBSS (20 mmol/L HEPES, 116 mmol/L NaCl, 12.5 mmol/L NaH2PO4, 5.6 mmol/L glucose, 5.4 mmol/L KCl, 0.8 mmol/L MgSO4; pH 7.35) and added to the discontinuous gradient consisting of 40.5% and 58.5% Percoll in HBSS. After centrifugation at 1,400 × g for 30 min at 15°C, the cardiomyocytes were collected from the interface of the discontinuous Percoll gradient and further enriched by preplating for 60 min on noncoated dishes. Unattached cells were cultured as cardiomyocytes in medium 199 with 10% FBS. NRCMs were infected with adenovirus vectors at multiplicity of infection of 20 for 2 hr [38]. Stealth small interfering (si)RNA targeting rat high-MW Gab1 was synthesized by Invitrogen. The nucleotide sequences of high-MW Gab1 #1 and #2 siRNAs were as follows: #1 siRNA, 5’-GAGAGGAGUACCUACUACUGGAAGA-3’ (sense) and 5’-UCUUCCAGUAGUAGGUACUCCUCUC-3’ (antisense); #2 siRNA, 5’-GAAGAAGGAGAGGAGUACCUACUAC-3’ (sense) and 5’GUAGUAGGUACUCCUCUCCUUCUUC-3’ (antisense). A nonspecific control siRNA from Invitrogen was used as a negative control. NRCMs were transfected with 10 nmol/L siRNA duplexes using Lipofectamine RNAiMAX reagent (Invitrogen) according to the manufacturer’s instructions. After incubation for 48 hr, the cells were used for the experiments.

After serum-starvation in medium 199 for 24 h, NRCMs were stimulated with 20 ng/mL NRG-1β for the indicated periods. Cells were lysed, precleared, and subjected to immunoprecipitation and immunoblot analysis following standard procedures as described earlier.

### Statistical analysis

Mean values, standard error of measurement, and statistical analysis for all data shown were calculated and plotted using GraphPad Prism 5 (GraphPad Software). All data were expressed as mean ± SEM. Differences among multiple groups were compared by one-way ANOVA followed by a post hoc comparison tested with Tukey’s method. A value of *P* < 0.05 was considered as statistically significant.

## Result

### High-MW Gab1 is specifically expressed in the heart

**Fig 1:**
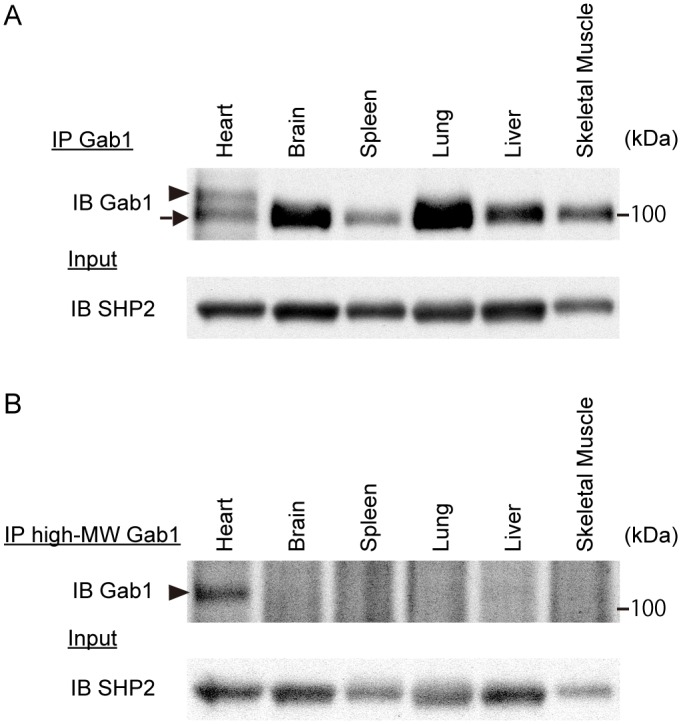
High-MW Gab1 is expressed in the heart. Expression patterns of A) Gab1 and B) high-MW Gab1 protein in mouse tissues. Following immunoprecipitation with A) anti-Gab1 or B) anti-high-MW Gab1 serum, expression of Gab1 was examined by immunoblotting analysis with anti-Gab1 antibody. The high-MW Gab1 is expressed in heart (arrowheads), and the low-MW Gab1 is commonly expressed (arrow). SHP2 was examined as a loading control. Results are representatives of three independent experiments.

To address the expression pattern of Gab1 in the various organs in mice, we performed immunoprecipitation and immunoblot analysis using the protein extracts from heart, brain, spleen, lung, liver, and skeletal muscle from C57BL/6J 8-wk old male mouse. Although low-MW Gab1 was detected in all the organs examined, high-MW Gab1 was exclusively expressed in the heart (Fig 1A). This finding is consistent with the data which we reported previously [22].

### High-MW Gab1 cDNA contains one skipped exon encoding highly conserved 27 amino acid residues

**Fig 2:**
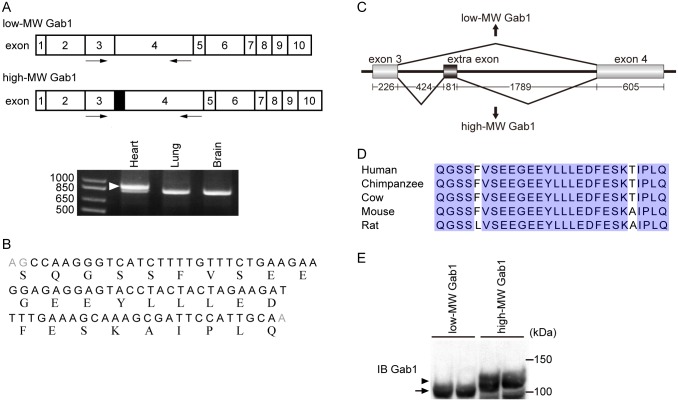
High-MW Gab1 contains extra-exon coding 27 amino acid residues. A) Mouse heart, lung, and brain RNA were subjected to RT-PCR using the forward primer to exon 3 (rightwards arrow) and the reverse primer to exon 4 (leftwards arrow). The long fragment (arrowhead) was detected in heart, but not in lung and brain. B) Nucleotide and amino acid sequence of the extra-exon. The derived amino acid sequence is shown below nucleotides. C) Schematic illustration of genomic structure of the Gab1 locus around the extra exon. D) Comparison of amino acid sequence of extra-exon from different species. Conserved amino acids in all of the species are highlighted in blue. E) Amino acid residues induce band-shift in western blot analysis. HEK293 cells were transfected with cloned low-MW Gab1 or high-MW Gab1. Cell lysates were collected and subjected to immunoblotting analysis with anti-Gab1 antibody. The arrowhead and arrow indicate the high-MW Gab1 and the low-MW Gab1, respectively.

We hypothesized that a skipped extra exon exists in the high-MW Gab1 cDNA. We systematically searched for an extra exon between the already-known exons of the low-MW Gab1 cDNA by comparing the RT-PCR products derived from the total RNAs of various organs. When using the forward primer in exon 3 and the reverse primer in exon 4, we found two distinct RT-PCR products in the RNAs from heart, but not in those from brain and lung (Fig 2A). On the other hand, we could not detect other extra exons in the heart RNA when using other combination of primers targeting other exons (S1 Fig and S1 Table). We found that a skipped extra exon between the already-known exon 3 and the exon 4 consists of 81 base pairs and encodes 27 amino acid residues (Fig 2B and 2C). Intriguingly, the 27 amino acid sequence encoded by this extra exon is highly conserved in mammals, suggesting that it might have a role for cardiomyocytes (Fig 2D). However, the 27 amino acid sequence does not show homology to the already-known functional domains.

We next isolated the mouse full-length cDNA (high-MW Gab1) containing the extra exon. HEK293 cells were transfected with the pcDNA3 vector containing the cDNAs encoding the low-MW and the high-MW Gab1, and we confirmed that the expected difference in electrophoretic mobility between the high-MW Gab1 and the low-MW Gab1 by western blot analysis (Fig 2E).

### High-MW Gab1 might be a novel striated muscle-specific Gab1 isoform

**Fig 3:**
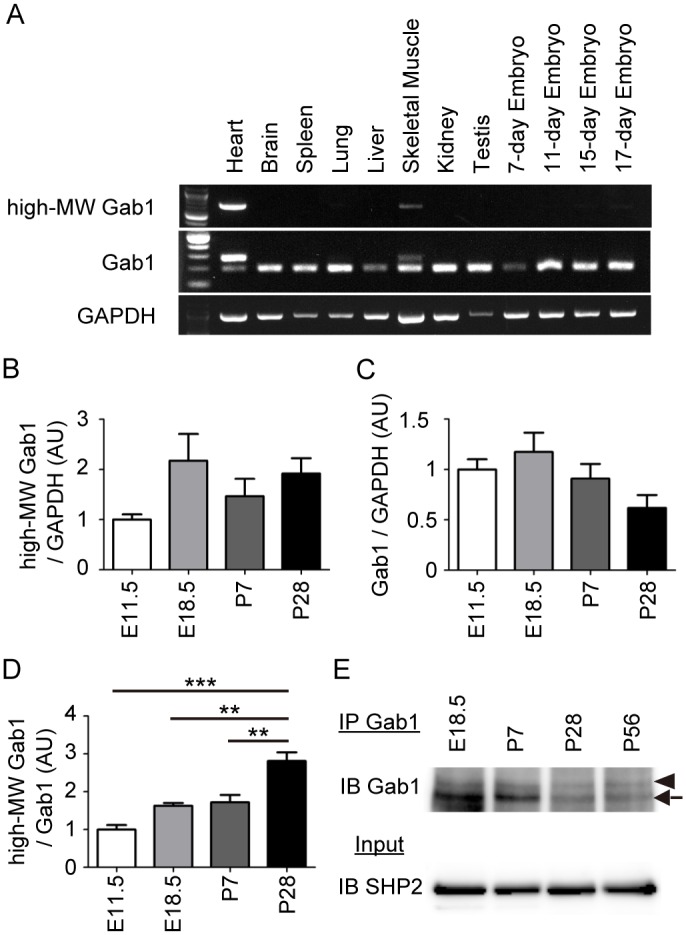
High-MW mRNA is expressed in striated-muscle. **A)** Expression pattern of Gab1 mRNA in mouse tissues and embryos. The primers for the high-MW Gab1 (top panel), Gab1 (middle panel), or GAPDH (control, bottom panel) were used to amplify cDNA from mouse tissues and embryos. B) and C) Quantitative expression analysis of *high-MW Gab1* (B) and *Gab1* (C) mRNAs in hearts at E11.5, E18.5, P7, and P28. (normalized to *GAPDH* mRNA; n = 3). Values are shown as mean±SEM for 3 separate experiments. One-way ANOVA followed by Tukey’s test was used to analyses differences. D) Quantitative expression analysis of *high-MW Gab1* to *Gab1* mRNA ratio in hearts at E11.5, E18.5, P7, and P28 (n = 3). Values are shown as mean±SEM for 3 separate experiments. One-way ANOVA followed by Tukey’s test was used to analyses differences. E) Expression patterns of high-MW Gab1 (arrowhead) and low-MW Gab1 (arrow) protein in mouse hearts at E18.5, P7, P28, and P56. Following immunoprecipitation with anti-Gab1 serum, expression of Gab1 was examined by immunoblotting analysis with anti-Gab1 antibody. SHP2 was examined as a loading control. Representative blots of 2 experiments are shown.

To determine the tissue expression pattern of the high-MW Gab1, we performed RT-PCR analysis using the cDNA panel derived from several adult mouse tissues and mouse embryos at various developmental stages. We performed two types of RT-PCR, using (1) the primers targeting the extra exon exclusively, and (2) the primers targeting the exon 3 and the exon 4. Both experiments showed that high-MW Gab1 was most strongly expressed in the adult heart and barely detected in adult skeletal muscle (Fig 3A). We next examined the expression level of Gab1 in mice hearts from embryonic to adult stage. Although the expression levels of *high-MW Gab1* and *Gab1* (including both of *high-* and *low-MW Gab1*) mRNA were not significantly different between embryonic and adult hearts, the *high-MW Gab1* mRNA to total *Gab1* mRNA ratio significantly increased along with heart development (Fig 3B–3D). We also performed immunoprecipitation and immunoblot analysis using the protein extracts from embryonic, postnatal, and adult mice hearts. Consistent with the qRT-PCR data, the high-MW Gab1 protein was expressed in the same level from embryonic to adult stage, although the expression level of the low-MW Gab1 protein decreased along with heart development (Fig 3E).

**Fig 4:**
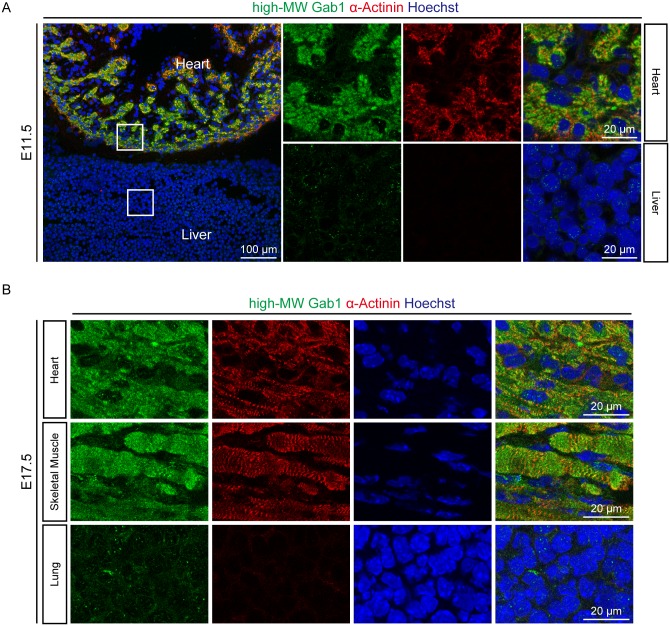
High-MW Gab1 is expressed in striated muscles of mouse embryo. A) and B) Immunohistochemical analyses of the high-MW Gab1 expression in mouse embryos. Sagittal sections of mouse embryos at E11.5 (A) and horizontal sections of mouse embryos at E17.5 were immunostained for the high-MW Gab1 (green) and α-Actinin (red). Representative images of 5 experiments (A) and 3 experiments (B) are shown.

Next, we raised rabbit polyclonal antibody specific for the high-MW Gab1. Immunostaining with this antibody in E11.5 embryos showed that the high-MW Gab1 was expressed in heart, but not in liver (Fig 4A). E17.5 embryos also showed that the high-MW Gab1 was expressed in heart and skeletal muscle, but not in lung (Fig 4B). We also performed immunoprecipitation and immunoblot analysis using the protein extracts from C57BL/6J 8-wk old male mice. The high-MW Gab1 was immunoprecipitated with this antiserum exclusively in the lysate extracted from heart, but not from other organs including skeletal muscles of adult mice (Fig 1B). These findings suggest that the high-MW Gab1 might be a novel striated muscle-specific Gab1 isoform.

### High-MW Gab1 undergoes tyrosine-phosphorylation in the murine heart after intravenous administration of NRG-1

**Fig 5:**
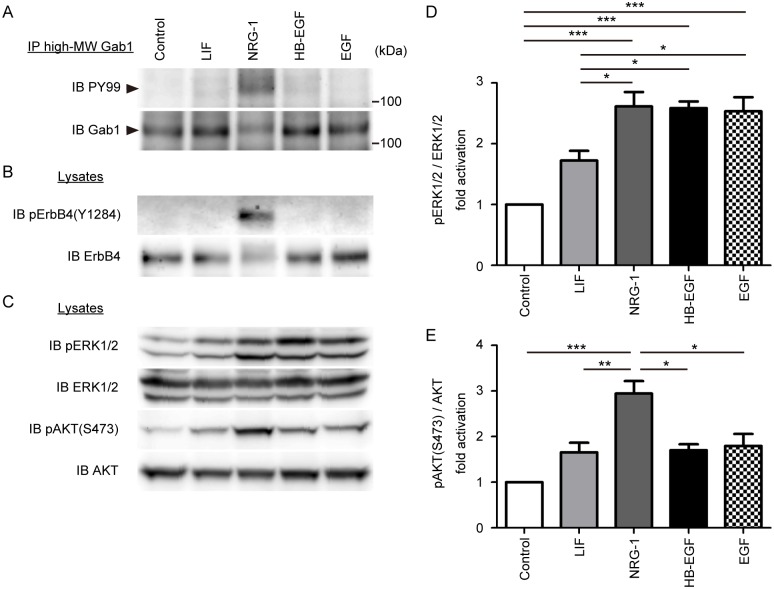
High-MW Gab1 is exclusively activated after stimulation with NRG-1 in heart. A) Tyrosine phosphorylation of the high-MW Gab1 was analyzed by immunoprecipitation of the heart lysates. Mouse heart lysates were prepared at 5 min after injection with the cytokines and growth factors listed at top. Heart lysates were subjected to immunoprecipitation with anti-high-MW Gab1 serum, followed by western blot analysis using the antibodies indicated at the left. Arrowheads denote the high-MW Gab1. B) Activation level of ErbB4 was assessed by phospho-specific antibody. C) Activation levels of ERK1/2 and AKT were assessed by phospho-specific antibodies. Heart lysates were subjected to western blot analysis with the indicated antibodies. Representative blots of 3 experiments are shown. D) Phosphorylation of ERK1/2 was quantified against total ERK1/2 (n = 3). E) Phosphorylation of AKT on Ser-473 was quantified against total AKT (n = 3). Values are shown as means±SEM for 3 separate experiments. One-way ANOVA followed by Tukey’s test was used to analyze differences. *P<0.05, **P<0.01, and ***P<0.001 for the indicated groups.

We previously reported that the high-MW Gab1 underwent tyrosine-phosphorylation exclusively upon stimulation with NRG-1 among several growth factors and cytokines [22]. To confirm the tyrosine-phosphorylation of the high-MW Gab1 in the adult murine hearts, we intravenously administrated several humoral factors such as leukemia inhibitory factor (LIF), NRG-1, HB-EGF, and EGF into wild-type mice. We subjected the heart lysates to immunoprecipitation with anti-high-MW Gab1 and western blot analysis. Consistent with previous data, the high-MW Gab1 underwent tyrosine-phosphorylation exclusively after administration of NRG-1, but not with LIF, HB-EGF, and EGF (Fig 5A) [22]. We also checked the activation of ErbB4. NRG-1 exclusively induced tyrosine-phosphorylation of ErbB4 (Fig 5B). Whereas activation of ERK1/2 was almost comparable in the mice treated with NRG, HB-EGF (Fig 5C and 5D), and EGF, activation of AKT was most strongly induced by NRG-1 (Fig 5C and 5E).

### High-MW Gab1 plays a crucial role in AKT activation downstream of NRG-1/ErbB signaling

**Fig 6:**
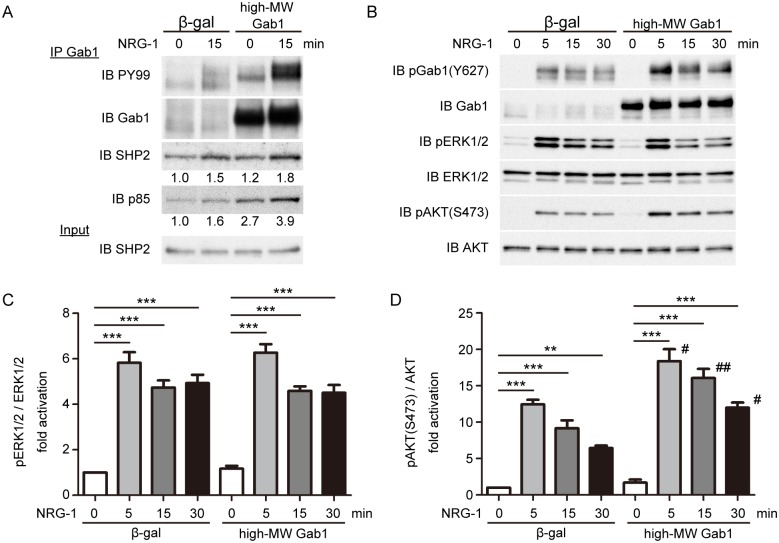
Forced expression of high-MW Gab1 enhances activation of AKT in response to NRG-1 in NRCMs. A) Tyrosine phosphorylation of Gab1 and its association with p85 and SHP2 were analyzed by immunoprecipitation of the NRCMs lysates. NRCMs, infected with the indicated adenovirus vectors, were stimulated with NRG-1 (20 ng/ml) for indicated time periods and cell lysates were subjected with immunoprecipitation with anti-Gab1 serum, followed by immunoblotting analysis using the antibodies indicated at the left. Values are expressed relative to the control group. Representative blots of 2 experiments are shown. B) Phosphorylation of Gab1, ERK1/2, and AKT were assessed by phosphor-specific antibodies. Representative blots of 4 experiments are shown. C) Phosphorylation of ERK1/2 was quantified against total ERK1/2 (n = 4). D) Phosphorylation of AKT on Ser-473 was quantified against total AKT (n = 4). Values are shown as means±SEM for 4 separate experiments. One-way ANOVA followed by Tukey’s test was used to analyze statistical differences. **P<0.01, ***P<0.001 for the indicated groups. #P<0.01, ##P<0.001 vs β-gal expressing cells at the same time after stimulation.

Next, we examined the role of the high-MW Gab1 in NRG-1/ErbB signaling. Since PI3-kinase pathway has been reported to have critical roles for the proliferation of the cardiomyocytes and protection by NRG-1/ErbB signaling [15,39], we focused on the activation of AKT after stimulation with NRG-1 in cardiomyocytes. First, we examined the effect of adenovirus-mediated forced expression of high-MW Gab1 on the NRG-1-dependent downstream signaling pathways. Overexpression of the high-MW Gab1 induced much stronger association of Gab1 with p85 than that with SHP2 upon stimulation with NRG-1 (Fig 6A). NRG-1 induced activation of AKT and ERK1/2 in the control NRCMs expressing β-galactosidase (β-gal) (Fig 6B). Whereas NRG-1-induced activation of ERK1/2 was not significantly changed in NRCMs expressing the high-MW Gab1 compared with control cells expressing β-gal (Fig 6B and 6C), activation of AKT was significantly enhanced in NRCMs expressing the high-MW Gab1 compared with control cells expressing β-gal (Fig 6B and 6D). In addition, NRG-1-induced activation of AKT was also enhanced in NRCMs expressing the low-MW Gab1 compared with control cells expressing β-gal, whereas activation of ERK1/2 was not significantly changed in NRCMs expressing the low-MW Gab1 compared with control cells expressing β-gal (S2 Fig). These findings suggest that the high-MW shares a role for enhancing the NRG-1-induced activation of AKT in NRCMs with the low-MW Gab1 isoform.

**Fig 7:**
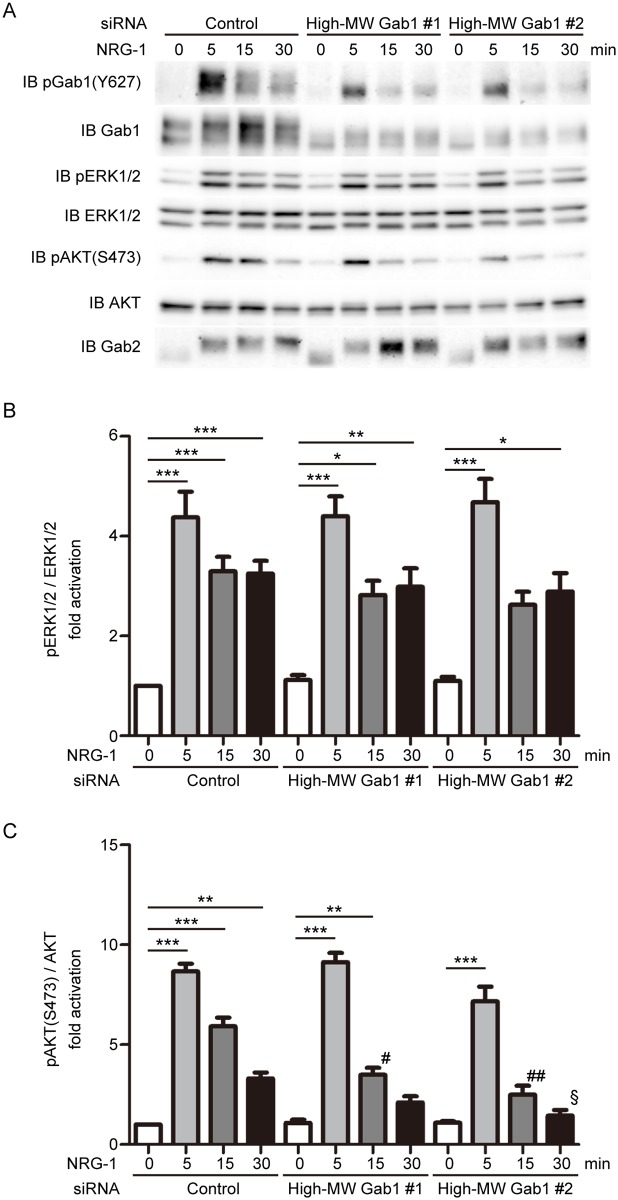
SiRNA-mediated knockdown of high-MW Gab1 attenuates sustained activation of AKT after NRG-1 stimulation in NRCMs. A) Representative images of western blot analysis. NRCMs, transfected with the indicated siRNA, were stimulated with NRG-1 (20 ng/ml) for indicated time periods. Cell lysates were collected and subjected to immunoblotting analyses using the antibodies indicated at the left. Representative blots of 5 experiments are shown. B) Phosphorylation of ERK1/2 was quantified against total ERK1/2 (n = 5). C) Phosphorylation of AKT on Ser-473 was quantified against total AKT (n = 5). Values are shown as means±SEM for 5separate experiments. One-way ANOVA followed by Tukey’s test was used to analyze differences. *P<0.05, **P<0.01, and ***P<0.001 for the indicated groups. §P<0.05, #P<0.01, ##P<0.001 vs β-gal expressing cells at the same time after stimulation.

Next, we performed siRNA-mediated knockdown of high-MW Gab1 in NRCMs. We observed successful depletion of the high-MW Gab1 protein in NRCMs at 72 hr after transfection with the high-MW Gab1-specific siRNA (Fig 7A). SiRNA-mediated knockdown of the high-MW Gab1 significantly attenuated sustained activation of AKT in response to NRG-1 in NRCMs (Fig 7A and C), but had no obvious effects on activation of ERK1/2 in response to NRG-1 (Fig 7A and 7B). Taken together, these results suggest that the high-MW Gab1 has a crucial role for AKT activation after stimulation with NRG-1 in NRCMs.

## Discussion

In the present study, we identified a novel isoform of Gab1 (high-MW Gab1). This high-MW Gab1 has a skipped exon coding 27 amino acid residues between the already-known 3^rd^ and 4th exons of the ubiquitously expressed low-MW Gab1. Whereas the high-MW Gab1 is expressed in both heart and skeletal muscle in embryos, it is exclusively expressed in heart during adulthood. The high-MW Gab1 in the heart undergoes tyrosine-phosphorylation only when stimulated with NRG-1. Forced expression of the high-MW Gab1 induces higher and more sustained activation of AKT after stimulation with NRG-1 compared with that of β-galactosidase in cardiomyocytes. Furthermore, knockdown of the high-MW Gab1 significantly attenuated NRG-1-induced activation of AKT in cardiomyocytes. These data indicate that the high-MW Gab1 isoform expressed in striated muscles might have a crucial role for activation of PI3-kinase/AKT pathway downstream of NRG-1/ErbB signaling in cardiomyocytes.

To our knowledge, there has been no report regarding a striated muscle-specific isoform of docking proteins. We identified a novel high-MW isoform (120 kDa) of Gab1 expressed exclusively in the striated muscles such as cardiac and skeletal muscles, in addition to the ubiquitously expressed low-MW (100 kDa) Gab1. In adult mice, the low-MW Gab1 is expressed ubiquitously in any cell types and in any organs [16–18,31]. It was reported that the expression of Gab1 is confined to myocardium from embryonic day 10.5 (E10.5) to E13.5, and becomes ubiquitous after E13.5 along with development [20]. The high-MW Gab1 isoform might contribute to the myocardium-specific expression pattern of Gab1 in midterm embryos and might be related to the cardiac defect in conventional Gab1KO mice [20].

Although Gab1 is a common docking protein mediating the signaling from diverse growth factors and cytokines, the high-MW Gab1 specifically underwent tyrosine-phosphorylation upon stimulation with NRG-1 (Fig 5A). We previously reported that intravenous administration of NRG-1 into C57BL6 mice induced tyrosine-phosphorylation of ErbB2 and ErbB4, but not that of ErbB1 and ErbB3 in the hearts [22]. In addition, we also showed that Gab1 associated with ErbB4 in NRG-1-dependent manner [22]. In the present study, we found that NRG-1 exclusively induced tyrosine-phosphorylation of ErbB4 among LIF, NRG-1, HB-EGF, and EGF (Fig 5B). These data indicate that NRG-1/ErbB4 signaling might specifically utilize Gab1 for activation of downstream pathways.

Forced expression of the high-MW Gab1 enhances and prolongs AKT activation after stimulation with NRG-1 in cardiomyocytes (Fig 6B–6D). On the other hand, overexpression of the low-MW Gab1 also enhances AKT activation after stimulation with NRG-1 in cardiomyocytes almost similarly as that of the high-MW Gab1 (S2 Fig). Whereas knockdown of the high-MW Gab1 significantly attenuated NRG-1-induced activation of AKT in cardiomyocytes (Fig 7), it was technically impossible to perform selective knockdown of the low-MW Gab1 in cardiomyocytes. Therefore, we could not exclude the possibility that the high-MW Gab1 and the low-MW Gab1 might share a similar role for activation of AKT downstream of NRG-1/ErbB signaling in cardiomyocytes.

In the previous study, we reported that cardiomyocyte-specific Gab1 knockout (Gab1CKO) was not sufficient to attenuate the activation of AKT upon NRG-1 administration [22]. The attenuation of AKT activation could only be achieved by Gab1 and Gab2 double knockout (DKO) [22]. For the creation of Gab1CKO, we crossed *alpha-MHC-Cre* transgenic mice with *Gab1flox* mice. In Gab1CKO and DKO mice, Gab1 protein expression was deleted in the cardiomyocytes from the embryonic stage, suggesting that some compensatory mechanisms might work. On the other hand, in the present study, we performed acute siRNA-mediated knockdown targeting high-MW Gab1 and found that the high-MW Gab1 knockdown attenuated the activation of AKT upon stimulation with NRG-1 in the neonatal rat cardiomyocytes. Therefore, the above discrepancy might be derived from the difference in experimental condition. Recent studies reported that Gab1CKO mice showed cardiac dysfunction under stressed condition such as myocardial ischemia/reperfusion injury and acute pressure overload with transaortic constriction [27,40]. Our findings implicate the possibility that Gab1CKO mice might show cardiac dysfunction under stressed condition due to impaired response to NRG-1.

In summary, we identified a novel high-MW Gab1 isoform which is exclusively expressed in heart and skeletal muscle, and characterized it as a crucial component for AKT activation downstream of NRG-1/ErbB-signalng in cardiomyocytes.

## Supporting Information

S1 Fig No other extra-exon was detected in the heart RNA. When we performed RT-PCR using forward primer to exon 1 and reverse primer to exon 3, forward primer to exon 4 and reverse primer to exon 5, or forward primer to exon 5 and reverse primer to exon 10, one fragment was detected in the heart, brain and lung total RNA. PCR primers used in S1 Figure were summarized in S1 Table.
(TIF)

S2 Fig Forced expression of low-MW Gab1 enhances activation of AKT in response to NRG-1 in NRCMs. A) Phosphorylation of Gab1, ERK1/2, and AKT were assessed by phosphor-specific antibodies. Representative blots of 5 experiments are shown. B) Phosphorylation of ERK1/2 was quantified against total ERK1/2 (n = 5). C) Phosphorylation of AKT on Ser-473 was quantified against total AKT (n = 5). Values are shown as means±SEM for 5 separate experiments. One-way ANOVA followed by Tukey’s test was used to analyze differences. *P<0.05, **P<0.01, ***P<0.001 for the indicated groups. §P<0.05 vs β-gal expressing cells at the same time after stimulation.
(TIF)

S1 Table Primer sequences used in S1 Fig. (TIF)
