# Supplementary figures and images for: Molecular Characterization of Striated Muscle-Specific Gab1 Isoform as a Critical Signal Transducer for Neuregulin-1/ErbB Signaling in Cardiomyocytes
Source: PLoS One. 2016 Nov 18;11(11):e0166710. doi: 10.1371/journal.pone.0166710 (PMC5115770; doi:10.1371/journal.pone.0166710)

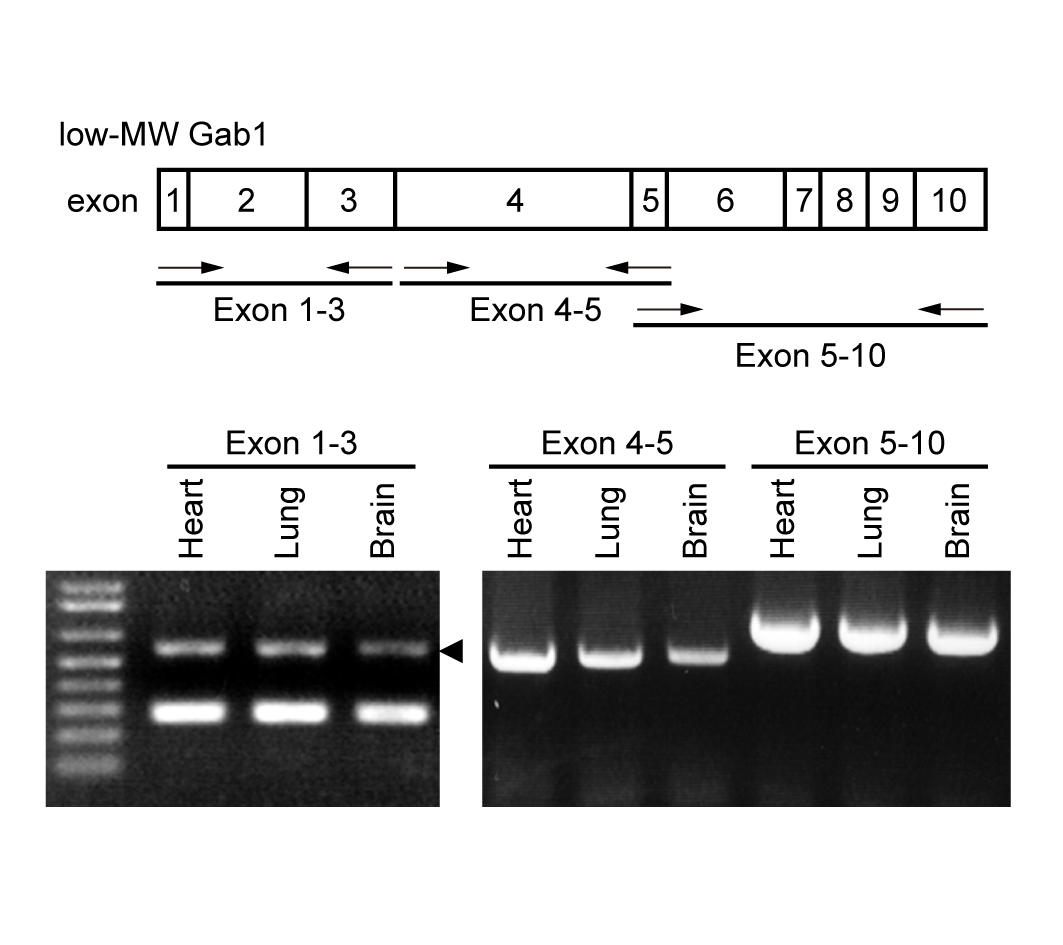

Supplement: S1 Fig — When we performed RT-PCR using forward primer to exon 1 and reverse primer to exon 3, forward primer to exon 4 and reverse primer to exon 5, or forward primer to exon 5 and reverse primer to exon 10, one fragment was detected in the heart, brain and lung total RNA. PCR primers used in S1 Figure were summarized in S1 Table. (TIF) [file pone.0166710.s001.tif]

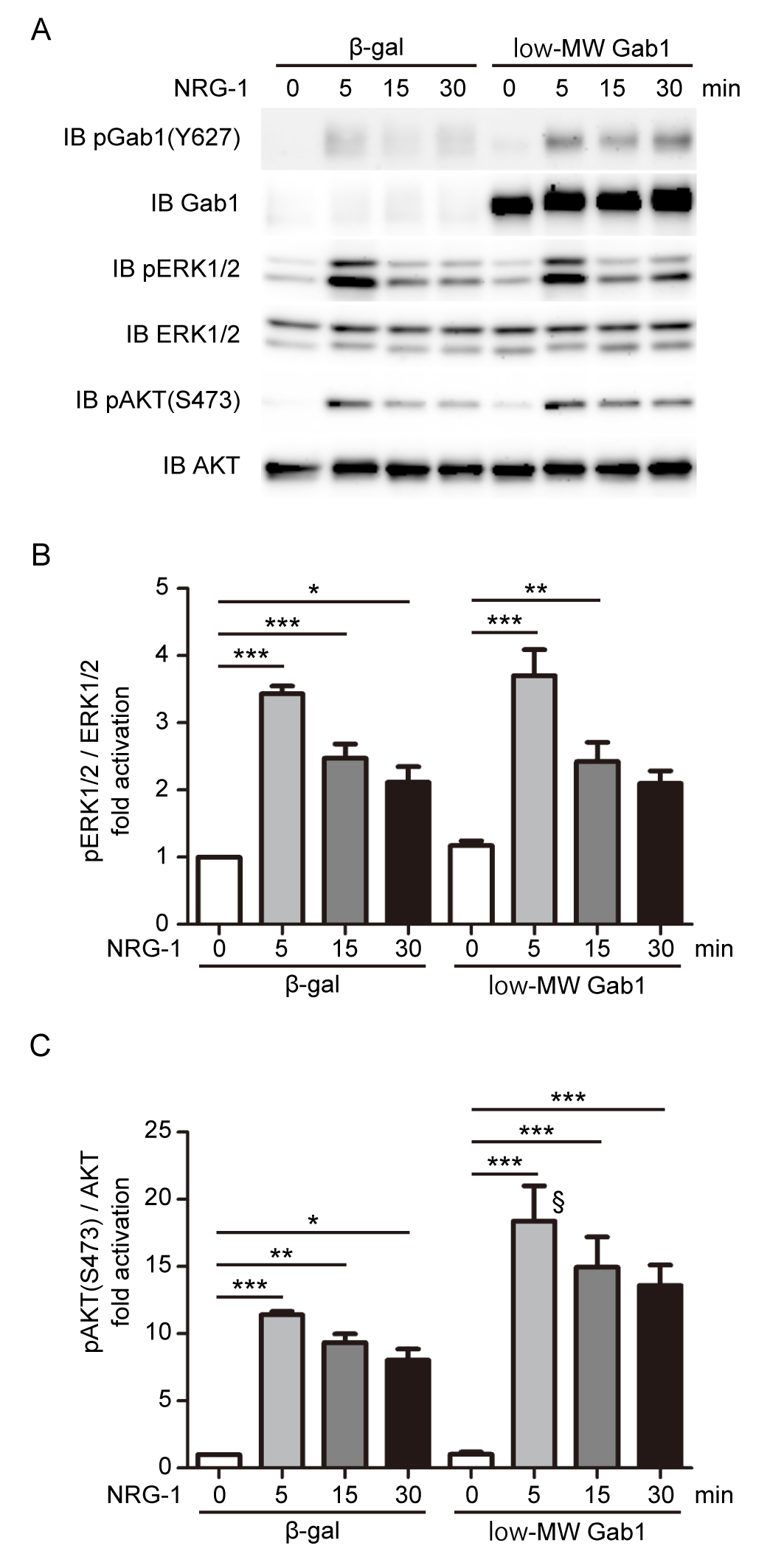

Supplement: S2 Fig — A) Phosphorylation of Gab1, ERK1/2, and AKT were assessed by phosphor-specific antibodies. Representative blots of 5 experiments are shown. B) Phosphorylation of ERK1/2 was quantified against total ERK1/2 (n = 5). C) Phosphorylation of AKT on Ser-473 was quantified against total AKT (n = 5). Values are shown as means±SEM for 5 separate experiments. One-way ANOVA followed by Tukey’s test was used to analyze differences. *P<0.05, **P<0.01, ***P<0.001 for the indicated groups. §P<0.05 vs β-gal expressing cells at the same time after stimulation. (TIF) [file pone.0166710.s002.tif]

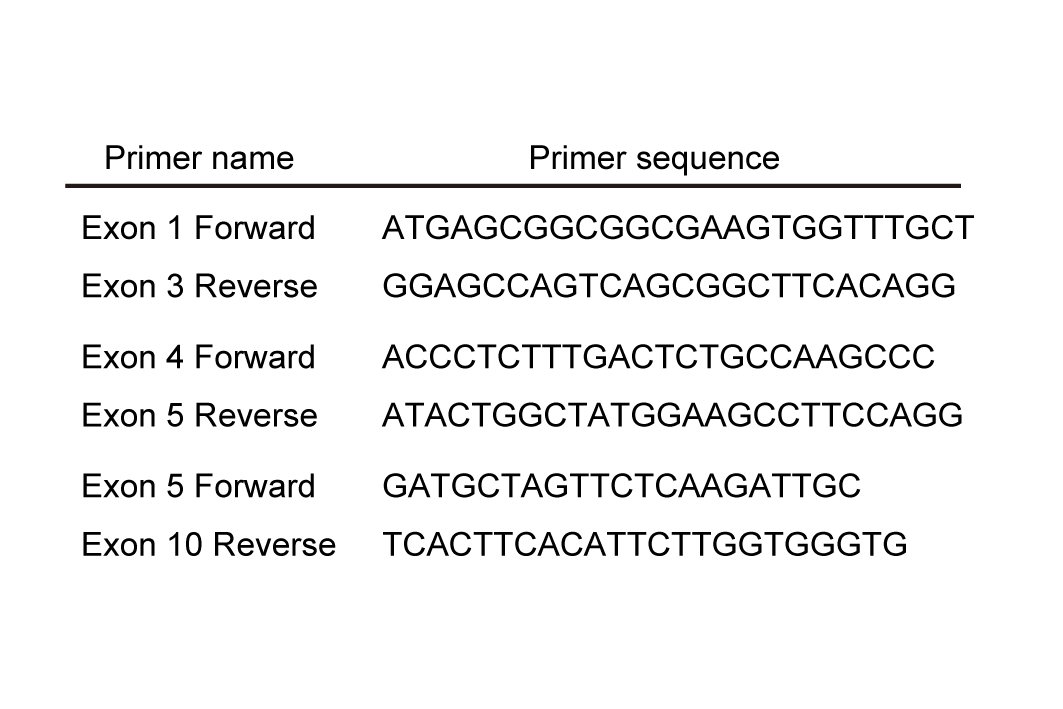

Supplement: S1 Table — (TIF) [file pone.0166710.s003.tif]
